# Carbon Dioxide Fixation Cascade for the Production of Single-Cell Protein by *Clostridium ljungdahlii* and *Yarrowia lipolytica*

**DOI:** 10.4014/jmb.2603.03009

**Published:** 2026-06-01

**Authors:** Gengjie Zhang, Mingchi Lai, Xuanyue Lu, Chuanzhao Wang, Fuli Li, Wenzhu Tang, Ziyong Liu

**Affiliations:** 1 School of Biological Engineering, Dalian Polytechnic University, Dalian 116034, P. R. China; 2 College of Environment and Safety Engineering, Qingdao University of Science and Technology, 53 Zhengzhou Road, Qingdao 266042, P. R. China; 3 Shandong Provincial Key Laboratory of Synthetic Biology, Key Laboratory of Biofuels, Qingdao Institute of Bioenergy and Bioprocess Technology, Chinese Academy of Sciences, No. 189 Songling Rd., Qingdao 266101, P. R. China

**Keywords:** *Clostridium ljungdahlii*, *Yarrowia lipolytica*, CO_2_ fixation, Single-cell protein, Integrated two-stage fermentation

## Abstract

*Clostridium* species and yeast single-cell protein (SCP), produced via industrial fermentation, offer a sustainable, high-protein alternative to conventional sources while advancing circular economy goals through CO_2_ valorization. Here, we developed an integrated two-stage bioprocess for SCP production from CO_2_. First, *Clostridium ljungdahlii* converted CO_2_ and H_2_ into SCP and acetate via gas fermentation. Optimizing nitrogen and sulfur feeding based on acetate synthesis rate increased acetate titer from 6 to 53.2 g/L and biomass from 1.1 to 2.8 (OD_600_). The fermentation broth was then directly used by *Yarrowia lipolytica*. Without pH control, dry cell weight (DCW) reached 4.7 g/L (67.0% protein, 6.5% lipid). With H_2_SO_4_-maintained pH 7, DCW reached 17.6 g/L (75.0% protein, 11.1% lipid). Notably, pH control using acetate yielded the highest DCW of 74.6 g/L (57.0% protein, 10.5% lipid). It should be noted that the highest biomass titer was achieved only when 130 g/L external acetate was added with pH control. This work demonstrates an efficient strategy for converting CO_2_ into valuable microbial protein, advancing scalable SCP production.

## Introduction

The escalating global demand for dietary protein, driven by population growth and changing consumption patterns, places immense strain on conventional agricultural systems. Traditional livestock production is associated with significant environmental burdens, including substantial land and water use as well as greenhouse gas emissions. This underscores an urgent need for sustainable, land-independent protein production alternatives. Single-cell protein (SCP), microbial biomass with high protein content produced via fermentation, represents a promising solution due to its high growth rate, independence from arable land, and consistent nutritional profile [1, 2].

Established SCP processes primarily utilize sugar-based feedstocks or C1-compounds like methanol. A more transformative approach is the direct biological conversion of carbon dioxide (CO_2_) into SCP, which aligns with circular bioeconomy and carbon-neutrality goals. Current routes for CO_2_-based SCP include the phototrophic cultivation of microalgae and the chemolithoautotrophic growth of hydrogen-oxidizing bacteria (HOB). However, these approaches present specific scalability or practical challenges. For microalgae, the low energy density of sunlight necessitates large cultivation areas. For HOB, technical complexities and safety risks are inherent in handling explosive H_2_/O_2_ gas mixtures [3, 4]. To address these limitations, we propose an alternative, integrated two-stage fermentation process for SCP production from gaseous substrates (CO_2_ and H_2_). This strategy employs a cascading carbon fixation approach. In the first stage, CO_2_ and H_2_ are biologically fixed by anaerobic acetogenic bacteria (*Clostridium ljungdahlii* in this study) into acetate and microbial biomass [5-8]. The acetate-rich fermentation broth from this clostridial stage is subsequently used as the primary carbon source to cultivate the oleaginous yeast *Yarrowia lipolytica*. This yeast is exceptionally suited for this role due to its natural ability to efficiently assimilate acetate, its robust growth under acidic conditions, and its generally recognized as safe status [9, 10]. *Y. lipolytica* produces biomass with a favorable nutritional profile, rich in both protein and lipids, rendering it a high-quality potential ingredient for animal feed [11, 12].

**Fig. 1:**
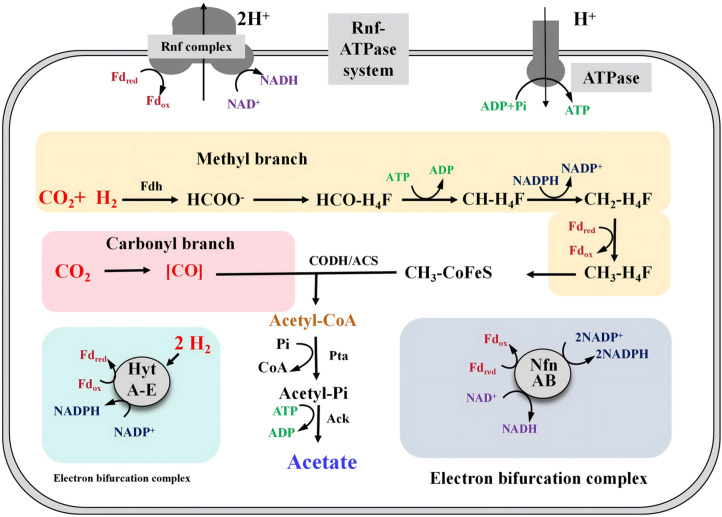
The acetate synthesis pathway in *Clostridium ljungdahlii* on CO_2_/H_2_.

Nevertheless, the efficiency of this coupled bioprocess is constrained by key technical factors. First, gas fermentation typically suffers from lower mass transfer efficiency compared to liquid fermentation, often making acetate production the rate-limiting step for overall biomass yield (Fig. 1) [13, 14]. Consequently, optimizing the gas fermentation stage is critical to enhancing CO_2_ fixation efficiency and downstream SCP productivity. Second, during yeast cultivation, the assimilation of acetate leads to a rise in the culture pH, requiring an effective control strategy to maintain optimal fermentation performance [15, 16]. This study therefore aims to explore an efficient pathway for SCP synthesis by systematically optimizing both fermentation stages. We focus on overcoming the mass transfer limitation in gas fermentation and implementing robust pH control during yeast cultivation on acetate, with the ultimate goal of improving the overall carbon conversion efficiency and protein yield of this integrated platform.

## Materials and Methods

### Microorganisms and Growth Conditions

*Clostridium ljungdahlii* DSM13528 used for the production of acetate, was purchased from the Deutsche Sammlung von Mikroorganismen und Zellkulturen GmbH, Braunschweig, Germany. The culture was stored at -80°C under anaerobic conditions and was transferred to a sterilized medium and activated at 30°C using a water bath before experiments. A previously engineered yeast *Y. lipolytica* po1g‐G3 was employed for acetate utilization and single-cell production [16, 17]. The culture was stored at -80°C in 20% glycerol solution and maintained regularly. Prior to experiments, the culture was thawed at room temperature and cultivated in a YPD medium comprising 20 g/L glucose, 20 g/L peptone, and 10 g/L yeast extract in a shaking incubator at 28°C and 250 rpm.

### Fermentation Media and Conditions

Fermentation PETC media (6 L) for acetate production by *C. ljungdahlii* comprised of 60 mL macronutrients stock, 6 mL trace elements stock, 1.2 g CaCl_2_, 4 mL vitamins, 3 g yeast extract powder, 0.6 g cysteine, and 0.12 g aminotriacetic acid. The macronutrients stock solution consisted of 100 g/L NH_4_Cl, 10 g/L KCl, 20 g/L MgSO_4_·7H_2_O, 80 g/L NaCl, 10 g/L KH_2_PO_4_, and 0.025 g/L Na_2_WO_4_·2H_2_O. Stock solution of trace elements contained 1.1 g/L MnSO_4_·H_2_O, 0.4 g/L FeSO_4_·7H_2_O, 0.2 g/L CoCl_2_·6H_2_O, 0.2 g/L ZnSO_4_·7H_2_O, 0.02 g/L NiCl_2_·6H_2_O, 0.2 g/L NaMoO_4_·2H_2_O, and 0.02 g/L Na_2_SeO_3_. The vitamin stock solution included 5 mg biotin, 2 mg folic acid, 10 mg vitamin B6, 5 mg thiamine, 5 mg riboflavin, 5 mg nicotinic acid, 8 mg calcium pantothenate, 5 mg cyanocobalamin, 5 mg para-aminobenzoic acid, and 5 mg lipoic acid. The gas fermentation was carried out in a 10-L fermenter (Zhenjiang Dongfang Bioengineering Equipment & Technology Co., Ltd., China) with a working volume of 6 L. The rotor speed was ranged between 200 and 500 rpm, and the gas was supplied at a flow rate of 20-0 mL/min. The temperature was controlled at 36°C and pH was controlled at 6.0 automatically by adding 4 M KOH. The yeast fermentation was carried out in in a 3-L fermenter (Dibio Bioengineering Equipment & Technology Co., Ltd. China) with a working volume of 1.5 L. The fermentation medium used contained supernatant from the first stage fermentation containing 5 g/L yeast powder and 5 g/L ammonium sulfate. The temperature was controlled at 28°C and pH was controlled at 7.0 automatically by adding acetate acid or H_2_SO_4_ (sulfuric acid). Dissolved oxygen levels were maintained above 10% of air saturation by adjusting the agitation speed and aeration rate. Samples were withdrawn periodically to monitor cell growth, substrate consumption, and product formation. All experiments were performed in duplicate.

### Analytical Methods

Growth of cells was monitored by measuring the optical density at 600 nm (OD_600nm_). Biomass was quantified using dry cell weight (DCW). SCP in this study refers to the total harvested microbial biomass, including residual *Clostridium* cells from the first stage and yeast cells from the second stage, without separation. Cell pellets from 15‐ml culture samples were washed twice with distilled water and centrifuged at 8,000 rpm for 10 min. Weight was determined after 24‐h freeze‐drying. Acetate, ethanol, and citrate were measured by an Agilent 1100 high-performance liquid chromatography (HPLC) and an Agilent Hi-Plex H column equipped with a refractive index detector operated at 35°C as previously established [18]. Samples were withdrawn every 12 h to measure OD and products. All the experiments were duplicated, and statistical analysis was performed using Microsoft Excel (2021), which was equipped with data analysis tools.

To extract total lipids, yeast cultures were centrifuged, and the wet cells were resuspended in 3 mL of 4 mol/L HCl in glass tubes and gently agitated for 90 min. After an 8-min boil, the cells were frozen at -20°C for 30 min. Subsequently, 6 mL of chloroform: methanol (1:1) was added to the tubes and centrifuged at 2,000 g for 10 min. The supernatant was transferred into a clean tube, and 3 mL of 0.15% NaCl was added and then centrifuged. Lastly, the supernatant was collected and dried with nitrogen gas using the blowing concentrator. Lipid content and composition were measured by gas chromatography (GC) using the method described by Su *et al*. 2023 [16, 19]. The content of and NH^4+^ in the broth and proteins in the cells were determined by the Kjeldahl method using the Kjeldahl instrument K9840 (Hanon Shandong Scientific Instruments Co., Ltd., China) according to the manufacturer's instructions. All experiments were performed in duplicate independent biological replicates (n = 2). For each replicate, technical triplicates were measured for analytical assays (*e.g.*, protein, lipid, acetate concentration). Error bars in all figures represent standard deviation (SD) calculated from the two independent biological replicates.

## Results and Discussion

### Effect of Sulfur and Nitrogen Source on the Growth and Acetate Synthesis

**Fig. 2:**
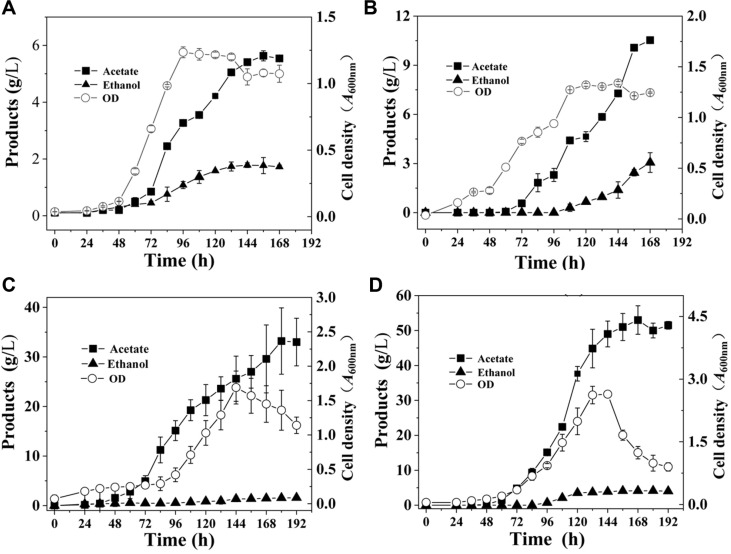
The growth and products of *C. ljungdahlii* grown on CO_2_/H_2_. (**A**) The pH control of gas fermentation with 4 M KOH; (**B**) Sulfur source (Na_2_S) added into KOH solution for pH control; (**C**) Nitrogen source (NH_4_OH) added into KOH solution for pH control; (**D**) Sulfur and nitrogen sources (Na_2_S+ NH_4_OH) added into KOH solution for pH control. Error bars in all figures represent standard deviation (SD) calculated from the two independent biological replicates.

In the gas fermentation process of *C. ljungdahlii*, the improvement in biomass and acetate production is closely associated with the supply of biological energy in the form of ATP. The transmembrane proton gradient serves as a critical driving force for ATP generation, making pH a key factor influencing ATP synthesis rate and, consequently, cell growth and metabolism [20-22]. A constant pH is conducive to studying high-concentration acetate fermentation. In this study, the pH was maintained at 6.0 throughout the fermentation process. Moreover, many key enzymes in the central metabolic pathway of *C. ljungdahlii* are metalloenzymes, necessitating the inclusion of various metal elements in the fermentation medium [23]. However, during medium preparation and sterilization, these metal ions tend to precipitate with other nutrients. Therefore, it is essential to carefully control the initial concentrations of these nutrients, which poses a challenge for achieving high acetate concentrations. To address this, we first conducted baseline fermentations with *C. ljungdahlii* under standard conditions at pH 6.0. As shown in Fig. 2A, after a one-day lag phase, the culture entered exponential growth, reaching a maximum biomass (OD_600_ = 1.2) at 96 hours. Subsequently, the OD decreased slowly. The maximum acetate titer reached 5.6 ± 0.2 g/L, accompanied by a small amount of ethanol (1.7 ± 0.3 g/L). The fermentation profile in Fig. 2A indicates a positive correlation between acetate accumulation and cell growth. However, both cell density and acetate production remained relatively low. Given the continuous gas supply, this limitation was attributed to nutrient deficiency rather than substrate limitation.

To overcome this constraint, we innovatively integrated nutrient supplementation with pH control by adding specific elements directly into the alkali solution used for pH adjustment. First, Na_2_S was incorporated into the 4 M KOH solution used for pH control at a concentration of 1 g per 400 mL of KOH. This allowed for the continuous addition of the sulfur source as acetate was produced, necessitating base addition. Fermentations conducted with this S-supplemented condition, while keeping other conditions identical, are shown in Fig. 2B. The results demonstrate a 66.7% increase in acetate titer, reaching 10.5 ± 0.2 g/L, compared to the control without sulfur source supplementation, while biomass increased only slightly. This confirms the crucial role of sulfur in maintaining the activity of key acidogenic enzymes; its addition promoted acetate synthesis. Concurrently, ethanol production increased substantially to 3.1 ± 0.6 g/L. Since ethanol synthesis requires more reducing equivalents than acetate production, this increase suggests that sulfur supplementation also enhanced the supply of reducing power during metabolism. Furthermore, doubling the Na_2_S concentration from 1 g to 2 g per 400 mL KOH solution did not significantly alter the final acetate yield (~11.3 ± 0.4 g/L) or ethanol yield (~3.5 ± 0.1 g/L), indicating that the sulfur source was already sufficient for the fermentation (Fig. S1). In addition, Na_2_S acts as a sulfide donor, a critical requirement for the activity of certain metalloenzymes in *C. ljungdahlii*, including CO dehydrogenase/acetyl-CoA synthase and hydrogenases. Sulfide preserves metal ions (*e.g.*, Fe, Ni, and Co) in a reduced and bioavailable form, thereby preventing their irreversible precipitation as insoluble metal oxides or hydroxides. Thus, these effects promote increased acetate and ethanol production.

Subsequently, a similar strategy was applied to nitrogen by incorporating ammonium hydroxide (NH_4_OH) into the alkali solution (composed of 350 mL 4M KOH and 50 mL 30% NH_4_OH per 400 mL), enabling the gradual addition of nitrogen as acetate was produced. This strategy yielded a more pronounced effect (Fig. 2C): acetate production surged to 33 ± 4.8 g/L, while ethanol production decreased. This indicates that sustained and balanced nitrogen supply not only promoted biomass growth (OD_600_ = 1.7) but also optimized carbon flux distribution, directing it primarily towards acetate synthesis. As a weak base, NH_4_OH contributes to stabilizing the pH of the culture medium. Furthermore, it serves as a source of ammonium ions, which are readily utilized for cell growth and protein synthesis. This, in turn, promotes higher biomass accumulation and increases the metabolic flux toward acetate in *C. ljungdahlii*. Notably, after 144 h, the OD of the strain decreased significantly, but acetate production continued. The addition of the nitrogen source promoted strain growth; however, it simultaneously accelerated the depletion of other key elements, leading to cell autolysis. Nevertheless, this result indicates that non-growing, metabolically active cells continue to synthesize acetate from gas, thereby increasing acetate concentration despite a decline in OD. Additionally, the release of intracellular acetate from autolyzed cells also contributed to the sustained increase in acetate production.

The most significant breakthrough was achieved through the combined and precise co-feeding of nitrogen and sulfur. When both elements were supplemented simultaneously via the alkali solution, a strong synergistic effect was observed: acetate production reached 53.2 ± 4.1 g/L, representing a 786.7% increase compared to fermentations without N and S sources supplementation (Fig. 2D). Biomass also increased substantially (OD_600_ = 2.65). During the entire fermentation process, 636 ± 33.7 mL of alkaline solution was consumed, which corresponds to an addition of 2.93 g/L of NH_4_^+^ to the 6 L fermentation system. The fermentation medium used in this study initially contained 1.2 g/L of NH_4_^+^. Considering that the starting medium also contained 0.3 g/L of NH_4_^+^, the results showed that a total of 2.03 g/L of NH_4_^+^ was consumed throughout the fermentation process. Under the current addition strategy, NH_4_^+^, as nitrogen source, was supplied in sufficient quantity. These results demonstrate that adequate nitrogen supply supports the synthesis of cellular materials and enzymes, while sufficient sulfur ensures the formation of active centers in key sulfur-containing enzymes of the Wood-Ljungdahl pathway. Together, the addition of Na_2_S and NH_4_OH ensures a stable, reduced environment with adequate metal bioavailability and pH control, thereby enabling sustained and increased acetate production and biomass accumulation.

In conclusion, the transition from simple pH control to the precisely synchronized supplementation of sulfur and nitrogen, particularly the combined N-S strategy, fundamentally improved acetate production by *C. ljungdahlii* through optimizing the microenvironment for cell growth and metabolism. To our knowledge, the use of automated pH control through the addition of key elements to alkaline solutions has not been reported as a means to increase biomass and product yield in gas fermentation. This integrated approach establishes a critical technological pathway for efficient acetate production from syngas fermentation. The core mechanism lies in matching the nutrient supply rate with the microbial metabolic rate, thereby avoiding the inhibitory effects often associated with traditional batch addition methods. Given that the addition of Na_2_S increases not only acetate production but also ethanol production, there is potential to further reduce the amount of Na_2_S without compromising acetate yield. This strategy lays a solid foundation for industrial scale-up. Future research could focus on further optimizing gas supply and fermentation parameters, combined with metabolic engineering of the strain, to achieve even higher productivity and economic viability.

### The Utilization of Gas Fermentation Broth for Yeast Cultivation

**Fig. 3:**
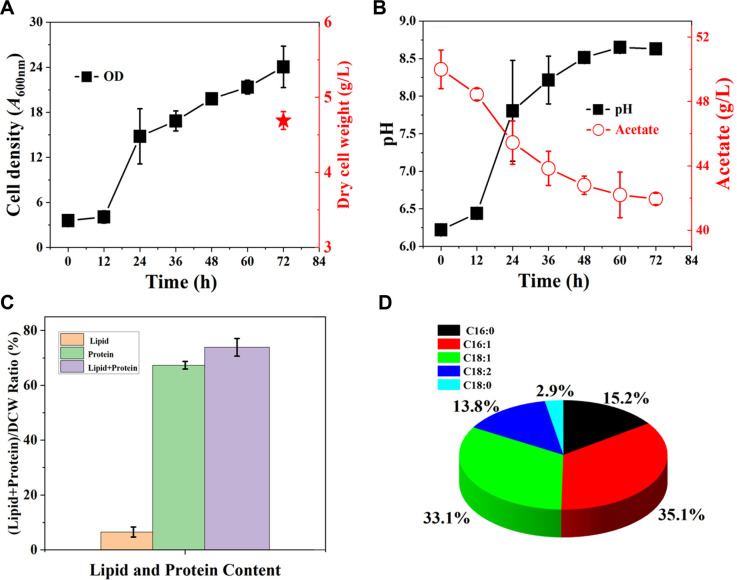
The growth and products of *Y. lipolytica* grown on gas fermentation broth without pH control. (**A**) DCW and OD measurement; (**B**) Acetate consumption and pH change; (**C**) Ratio of lipid and proteins in DCW; (**D**) Fatty acid composition in the lipid detected by gas chromatography. Error bars in all figures represent standard deviation (SD) calculated from the two independent biological replicates.

The non-purified and non-concentrated broth obtained from gas fermentation, following supplementation with yeast extract and ammonium sulfate, was directly transferred into a sterile aerobic bioreactor. Upon inoculation with *Y. lipolytica*, fermentation was conducted under controlled conditions to evaluate process performance. In the absence of pH control (Fig. 3), the pH increased progressively as acetate was consumed, reaching values above 8.0, which severely restricted yeast proliferation and metabolic activity. Maximal biomass was achieved at 72 h, with a cell density (OD_600nm_) of 24 ± 2.8 and a DCW of 4.7 ± 0.1 g/L. The protein and lipid contents accounted for 67% and 6.5% of DCW, respectively, cumulatively representing 73.9% of DCW. The fatty acid profile was predominantly composed of palmitic acid (C16:0), palmitoleic acid (C16:1), oleic acid (C18:1), linoleic acid (C18:2), and stearic acid (C18:0). Notably, the unsaturated fatty acids palmitoleic acid and oleic acid each comprised over 30% of total lipids, establishing them as the principal fatty acid constituents (Fig. 3D).

The amino acid profile of *Y. lipolytica* biomass revealed a favorable nutritional composition, with a high abundance of essential amino acids, particularly those commonly limiting in plant-based proteins [9]. Relative to *Saccharomyces cerevisiae*, *Y. lipolytica* exhibited approximately 50% higher alanine content, over 30% increases in lysine, isoleucine, and tryptophan, and more than 20% elevations in leucine, valine, glycine, and glutamic acid. Given that lysine is often the first limiting amino acid in cereal-based feeds, the elevated lysine content of *Y. lipolytica* confers a complementary nutritional advantage when combined with such substrates [10]. The fatty acid composition further supports its suitability as a protein feed supplement; unsaturated fatty acids (oleic acid, linoleic acid) are recognized for their superior health and growth-promoting properties relative to saturated fatty acids (palmitic acid, stearic acid). In *Y. lipolytica*, the proportion of unsaturated fatty acids was approximately 4.5-fold higher than that of saturated fatty acids, underscoring its potential as a high-quality feed protein source (Fig. 3D) [24, 25]. However, when cultivated directly in gas fermentation broth with acetate as the sole carbon source, the absence of pH control resulted in a pH exceeding 8.0, which inhibited yeast growth and metabolism, leading to inefficient acetiate utilization and diminished single-cell protein (SCP) yields [26].

**Fig. 4:**
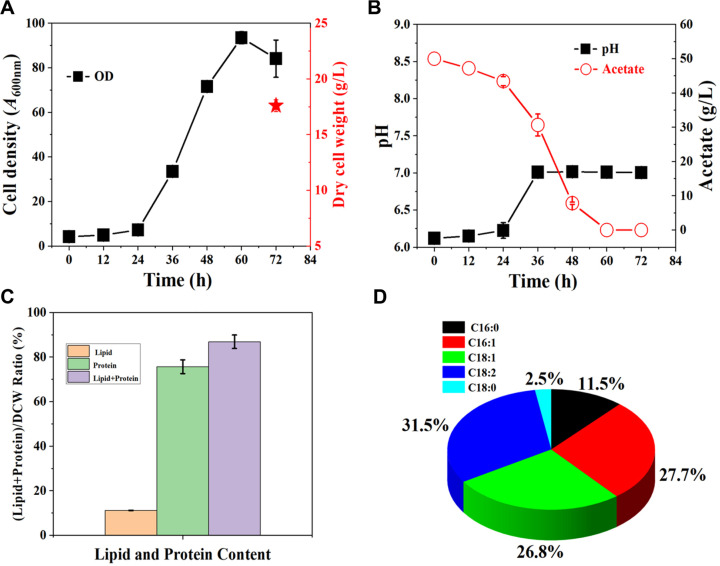
The growth and products of *Y. lipolytica* grown on gas fermentation broth with pH control (pH 7.0) by adding H_2_SO_4_. (**A**) DCW and OD measurement; (**B**) Acetate consumption and pH change; (**C**) Ratio of lipid and proteins in DCW; (**D**) Fatty acid composition in the lipid detected by gas chromatography. Error bars in all figures represent standard deviation (SD) calculated from the two independent biological replicates.

To enhance acetate assimilation, pH-controlled fermentations were conducted using H_2_SO_4_ to maintain the pH below 7.0 (Fig. 4). Following a 24 h lag phase, the culture entered a period of exponential growth (Fig. 4A and 4B). H_2_SO_4_ addition was initiated at approximately 36 h to sustain pH 7.0. Maximum biomass was attained at 60 h, with a cell density (OD_600nm_) of 93.5 ± 3.0, coinciding with complete depletion of acetate from the medium. Beyond this point, continued cultivation led to a decline in biomass. At fermentation termination, DCW was determined to be 17.6 ± 0.5 g/L (Fig. 4A). Protein and lipid contents reached 75% and 11.1% of DCW, respectively, collectively comprising 86.9% of DCW (Fig. 4C). The fatty acid profile remained consistent with previous observations, although linoleic acid (C18:2), a polyunsaturated fatty acid, emerged as the dominant component, accounting for 31.5% of total fatty acids (Fig. 4D). Compared to uncontrolled pH conditions, this strategy substantially improved biomass accumulation and DCW, with complete acetate consumption within 60 h and enhanced lipid and protein contents. Nevertheless, the use of H_2_SO_4_ for pH control resulted in significant sulfate accumulation in the post-harvest broth, potentially increasing downstream wastewater treatment costs during scale-up.

**Fig. 5:**
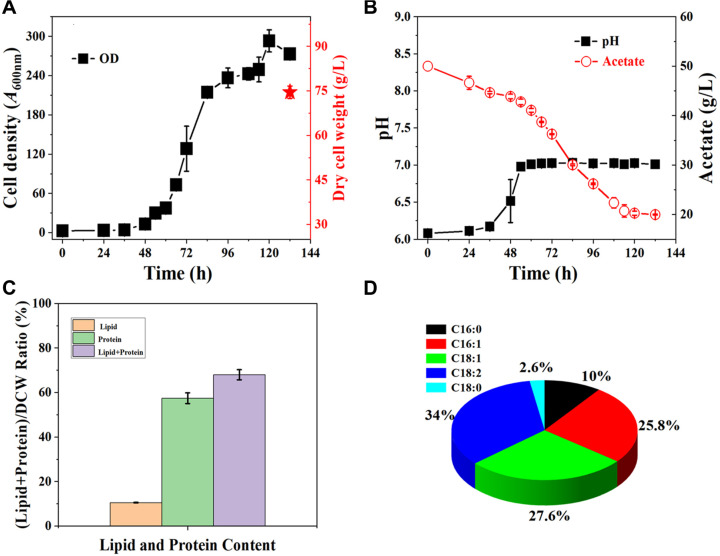
The growth and products of *Y. lipolytica* grown on gas fermentation broth with pH control (pH 7.0) by adding acetate. (**A**) DCW and OD measurement; (**B**) Acetate consumption and pH change; (**C**) Ratio of lipid and proteins in DCW; (**D**) Fatty acid composition in the lipid detected by gas chromatography. Error bars in all figures represent standard deviation (SD) calculated from the two independent biological replicates.

To circumvent sulfate accumulation in the broth, pH control was implemented using acetate, replicating the fermentation process under otherwise identical conditions (Fig. 5). A prolonged lag phase of 36 h preceded exponential growth. Acetate addition for pH maintenance commenced at 54 h (Fig. 5B). Despite continuous supplementation, acetate concentration declined slowly, and fermentation ceased at 120 h when consumption halted, leaving approximately 20 g/L residual acetate. Maximum biomass reached a cell density (OD_600nm_) of 293 ± 17, with a DCW of 74.6 ± 2 g/L (Fig. 5A). Protein and lipid contents were 57% and 10.5% of DCW, respectively, together accounting for 68.0% of DCW (Fig. 5C). The fatty acid profile remained qualitatively similar, with linoleic acid again predominant at 34% of total fatty acids (Fig. 5D). Although this strategy yielded substantially higher biomass and DCW due to the abundant carbon supply from acetate supplementation, the proportional yields of protein and lipids were notably lower than those achieved under the previous two conditions (Figs. 3C-5C). Given that the primary objective was SCP and lipid production, this reduction in target product content renders the acetate-mediated pH control strategy economically suboptimal. The observed decline in protein and lipid proportions suggests metabolic redirection toward alternative products. Notably, 0.3 g/L citrate was detected in the broth under acetate-controlled conditions, a metabolite absent in the uncontrolled and H_2_SO_4_-controlled fermentations. This finding indicates a shift in intracellular carbon flux away from protein and lipid synthesis toward organic acid production or other metabolites, consistent with previous reports of citrate accumulation in *Y. lipolytica* under specific cultivation regimes. Importantly, acetate consumption calculations revealed that approximately 130 g/L of acetate was supplemented via pH control, far exceeding the initial broth concentration. This externally added acetate originated from an external feed rather than the preceding gas fermentation stage, indicating a decoupling of the yeast fermentation from the upstream gas fermentation process.

Comparative evaluation of the three fermentation strategies demonstrates that H_2_SO_4_-mediated pH control achieves effective coupling with gas fermentation and yields SCP and lipid profiles aligned with research objectives (Table 1). Based on the fermentation data, the acetate-to-SCP carbon conversion efficiency can be roughly estimated. In the yeast fermentation, the acetate-rich broth was used without prior separation, producing 17.6 g/L DCW. Excluding the carbon contribution from yeast extract and residual *Clostridium* cells, nearly all carbon in the yeast biomass is assumed to derive from acetate metabolism. Assuming a carbon content of 50% of yeast DCW, the carbon in harvested yeast was 8.8 g/L. Acetate (CH_3_COOH, MW = 60.05) contains 40% carbon by mass. Therefore, 53.2 g/L acetate corresponds to 21.28 g/L of carbon. Thus, the overall carbon conversion efficiency from acetate to yeast dry weight (SCP) is 41.4% (8.8/21.28*100%).

However, the current results show that acetate production via gas fermentation remains the rate-limiting step in the overall carbon fixation pathway for SCP production. Therefore, addressing this limitation will require deeper integration of the two fermentation processes to further enhance the efficiency of acetate production from gaseous substrates. Additionally, the residual sulfate in the fermentation broth presents a challenge for downstream wastewater treatment, necessitating further process optimization or the integration of alternative technologies to address this limitation.

### A Comparative Analysis of CO_2_-Fixation Systems

CO_2_-fixation studies using a variety of approaches in microbial carbon capture and utilization, including different microorganisms, fermentation conditions, and end products, are presented. The fermentation strategies employed in these studies include batch, fed-batch, and continuous processes. Continuous gas fermentation is common in studies using gaseous substrates. In addition, fed-batch fermentation is utilized in studies aiming for high product concentrations. Bacterial species such as *C. ljungdahlii*, *C. autoethanogenum*, *Ralstonia eutropha*, *Acetobacterium woodii* and *Moorella thermoacetica* are prominent in several studies due to their natural CO_2_-fixing abilities. For example, Abubackar *et al*. demonstrated ethanol production (0.9 g/L) from CO and syngas using *C. autoethanogenum* in a continuous gas fermentation system [27]. Several studies have explored the use of CO_2_ as the carbon source for butanol and hexanol production by *C. carboxidivorans*. In a notable variation, Phillips *et al*. cultivated the bacterium in a nutrient-limited medium devoid of yeast extract and with minimal complex chemical components, using syngas (a mixture of CO_2_, H_2_, and CO) as the substrate to produce ethanol, butanol, and hexanol [28]. Another study by Liu *et al*. is distinctive in that it uses an electrochemical system to supply electrons for CO_2_ fixation in engineered *Ralstonia eutropha*, resulting in isopropanol production with a 50% energy efficiency in CO_2_ reduction [29]. In a batch fermentation conducted in a stirred-tank reactor with continuous sparging of H_2_ and CO_2_ under pH-controlled conditions (pH 7), acetate production by genetically modified strains of *A. woodii* was significantly enhanced. Final acetate concentrations exceeding 50 g L^-1^ were achieved in less than four days under autotrophic conditions, despite low cell densities of only 1.5–2 g L^-1^ DCW [30]. These bacterial cells have the potential to serve as single-cell protein (SCP); however, its biomass concentration remains low during CO_2_ fermentation, posing a bottleneck for SCP production. A substantial portion of carbon is directed toward the synthesis of byproducts such as acetate and ethanol, which not only complicates downstream separation but also renders the process unsuitable for SCP generation. A two-stage integrated bioconversion process was developed, in which *M. thermoacetica* first converts syngas into acetate, followed by the conversion of acetate into lipids by an engineered oleaginous yeast, *Y. lipolytica*. The integrated continuous reactor system enabled the production of 18 g/L of C16–C18 triacylglycerols from syngas [15]. Our study also presents a two-step cascade process combining *C. ljungdahlii* and *Y. lipolytica* to produce SCP. By optimizing the gas fermentation process, we not only enabled the production of single-cell protein (SCP) from *C. ljungdalii* but also enhanced acetate synthesis. Without prior separation, the acetate-rich fermentation broth was directly used to cultivate yeast, allowing the acetate generated during gas fermentation to support the production of a mixed microbial biomass comprising both *Clostridium* and yeast. Furthermore, this two-stage fermentation strategy can be operated in series with appropriate buffering and recycling, enabling a semi-continuous or continuous process for SCP production using CO_2_. This integrated approach substantially improves the conversion efficiency of CO_2_ into SCP, presenting an effective strategy for microbial protein production.

## Conclusion

This study establishes an integrated two-stage bioprocess for CO_2_ conversion into single-cell protein (SCP) by coupling *C. ljungdahlii* gas fermentation with *Y. lipolytica* cultivation. Synchronized nitrogen and sulfur source supplementation via pH control alkali solution dramatically enhanced acetate production, achieving 53.2 ± 4.1 g/L—a 786.7% increase over unsupplemented controls. For downstream processing, H_2_SO_4_-mediated pH control proved optimal, yielding 17.6 ± 0.5 g/L DCW with protein and lipid contents of 75% and 11.1%, respectively, while achieving complete acetate consumption within 60 h. The integrated N-S co-feeding strategy, together with H_2_SO_4_-based pH control during yeast cultivation, allows the utilization of non-purified, non-concentrated broth (with appropriate supplementation), enhancing overall conversion efficiency.

## Supplemental Materials

Supplementary data for this paper are available on-line only at http://jmb.or.kr.



## Figures and Tables

**Table 1:** The acetate consumption and products concentrations in *Y. lipolytica* fermentation using gas fermentation broth

	Acetate Consumption^a^ (g/L)	Residual Acetate^b^ (g/L)	Final DCW (g/L)	Lipid Content (g/L)	Protein Content (g/L)	Fermentation Time (h)
Without pH control	11.4 ± 0.3	0	4.7 ± 0.1	0.42 ± 0.1	3.2 ± 0.1	72
pH control by H_2_SO_4_	52.3 ± 1.2	0	17.6 ± 0.5	1.97 ± 0.2	13.3 ± 0.3	60
pH control by Acetate	162.5 ± 2	20.0± 1.3	76.4 ± 2.0	7.9 ± 0.2	42.8 ± 2.4	120

^a^Acetate consumption in the whole fermentation process; ^b^The titer of acetate in the final broth; All experiments were performed in duplicate independent biological replicates (n = 2).
