# Supplementary material for: Carbon Dioxide Fixation Cascade for the Production of Single-Cell Protein by Clostridium ljungdahlii and Yarrowia lipolytica
Source: J Microbiol Biotechnol. 2026 Jun 1;36:e2603009. doi: 10.4014/jmb.2603.03009 (PMC13259618; doi:10.4014/jmb.2603.03009)
Supplement: Supplementary file 1 [file jmb-36-e2603009-supple.pdf]

J. Microbiol. Biotechnol. 2026. 36: e2603009

https://doi.org/10.4014/jmb.2603.03009

Carbon dioxide fixation cascade for the production of single-cell protein by *Clostridium ljungdahlii* and *Yarrowia lipolytica*

Gengjie Zhang[1,3,†], Mingchi Lai[2,3,†], Xuanyue Lu[3], Chuanzhao Wang[3], Fuli Li[3], Wenzhu Tang[1]* and Ziyong Liu [3]*

[1]School of Biological Engineering, Dalian Polytechnic University, Dalian 116034, China;
[2]College of Environment and Safety Engineering, Qingdao University of Science and Technology, 53 Zhengzhou Road, Qingdao 266042, P. R. China
[3]Shandong Provincial Key Laboratory of Synthetic Biology, Key Laboratory of Biofuels, Qingdao Institute of Bioenergy and Bioprocess Technology, Chinese Academy of Sciences, No. 189 Songling Rd., Qingdao 266101, P. R. China

* Correspondence: liuzy@qibebt.ac.cn; tangwenzhu2000@163.com;

* These authors are co-corresponding authors.

† These authors contributed equally to this work.

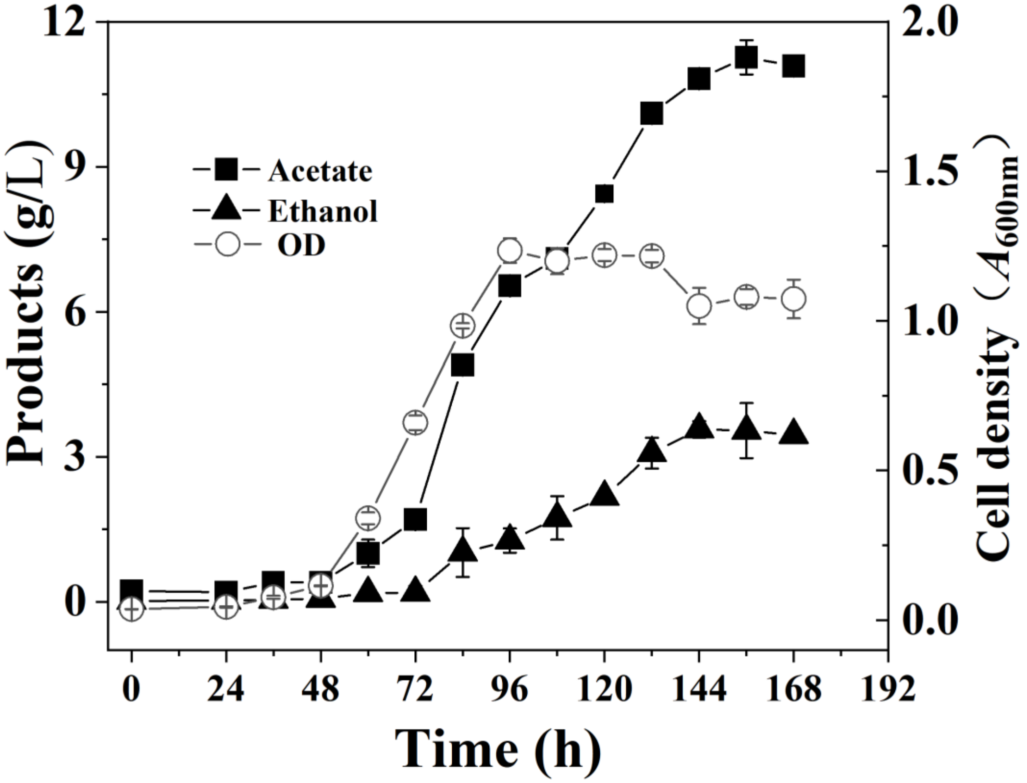

Fig. S1. The growth and products of *C. ljungdahlii* grown on CO_2/H_2 with Na_2S addition.
Twice the amount of sulfur source (2 g Na_2S) was added into 400 mL KOH solution for pH control. Error bars in all figures represent standard deviation (SD) calculated from the two independent biological replicates.
